# Enhancing Osteogenesis through Bio‐Inspired Recombinant Coral Protein Galaxin by Targeting Mitochondrial Metabolism and ATP Production

**DOI:** 10.1002/advs.202412867

**Published:** 2025-03-08

**Authors:** Fei Yu, Xing Zhao, Ting Jiang, Ningjuan Ouyang, Peilin Li, Wei Yang, Zhihe Zhao

**Affiliations:** ^1^ State Key Laboratory of Oral Diseases National Center for Stomatology National Clinical Research Center for Oral Diseases Department of Orthodontics West China Hospital of Stomatology Sichuan University Chengdu 610041 China; ^2^ Department of Nephrology Kidney Research Institute West China Hospital of Sichuan University Chengdu 610041 China; ^3^ Institute of Biomedical Engineering College of Medicine Southwest Jiaotong University Chengdu 610031 China; ^4^ Department of Pediatric Dentistry Shanghai Ninth People's Hospital Shanghai Jiao Tong University School of Medicine College of Stomatology Shanghai Jiao Tong University National Center for Stomatology National Clinical Research Center for Oral Diseases Shanghai Key Laboratory of Stomatology Shanghai 200011 China; ^5^ Department of Orthodontics Shanghai Ninth People's Hospital Shanghai Jiao Tong University School of Medicine College of Stomatology Shanghai Jiao Tong University National Center for Stomatology National Clinical Research Center for Oral Diseases Shanghai Key Laboratory of Stomatology Shanghai 200011 China; ^6^ College of Polymer Science and Engineering Med‐X Center for Materials Sichuan University Chengdu 610065 China

**Keywords:** bio‐inspired, bone regeneration, coral, galaxin, mitochondrial metabolism

## Abstract

Bio‐inspired coral‐derived scaffolds have been adopted for bone regeneration. However, these confront challenges such as the limited osteoinductive properties, environmental concerns, and ambiguous calcification mechanisms in coral proteins. In this study, the role of the recombinant coral protein galaxin in promoting osteogenesis is investigated. The observations reveal that recombinant galaxin regulates the mitochondrial metabolism in mouse pre‐osteoblasts by interacting with the β subunit of ATP synthase. This ultimately promotes osteogenesis of pre‐osteoblasts. Furthermore, galaxin is integrated with gelatin methacryloyl (GelMA) and assess the osteogenic potential of the resulting galaxin‐GelMA composites in mouse mandibular defects. The observations emphasize the distinctive role of galaxin in regulating mitochondrial functionality and osteogenesis. Additionally, these provide prospective insights for further applications of bio‐inspired recombinant coral proteins in regenerative medicine.

## Graphical abstract

The study highlights the potential of recombinant galaxin (a coral protein) as a novel and effective biomaterial for bone regeneration. It binds to ATP synthase subunit β (ATP5B) in mouse pre‐osteoblasts, thereby causing increased ATP production and mitochondrial activity. The galaxin‐conjugated gelatin methacryloyl (GelMA) composite shows potential bone regeneration capabilities in mouse mandibular defects. As a bioinspired alternative to conventional coral scaffolds, recombinant galaxin promotes sustainability and therapeutic considerations.

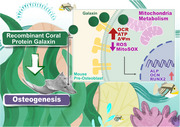

## 1 Introduction

The remarkable biomineralization capabilities of scleractinian corals, recognized as hypercalcifiers, are pivotal for extracting significant amounts of CaCO_3_ from seawater to form robust skeletons.^[^ ^1^, ^2^ ^]^ This process primarily occurs extracellularly at the interface between the underlying skeleton and the calicoblastic ectoderm, which is a single‐cell epithelial layer.^[^ ^3^ ^]^ Aragonitic CaCO_3_ consists of the supportive skeletons of scleractinian corals.^[^ ^4^ ^]^ The elucidation of coral calcification mechanisms could significantly advance the development of bone repair materials in tissue engineering.

Drawing inspiration from corals, the application of coral‐related scaffolds in bone tissue engineering has been promoted owing to their structural similarity to cancellous bone,^[^ ^5^, ^6^ ^]^ inherent porosity, load‐bearing capacity, and biodegradability.^[^ ^7^, ^8^ ^]^ Osteoinductivity and osteointegration are crucial advanced properties required for bone biomaterials.^[^ ^9^, ^10^ ^]^ However, not with standing the demonstration of biocompatibility, the ectopic implantation of coral materials has shown limited capacity to induce bone regeneration in the surrounding tissues.^[^ ^11^ ^]^ These osteoinductive properties can be enhanced by combining the coral scaffolds with mesenchymal stem cells^[^ ^12^, ^13^ ^]^ or growth factors.^[^ ^14^, ^15^ ^]^ Nevertheless, determining the preferable combination of coral species with appropriate mechanical strength and cellular compatibility is essential for effective application.^[^ ^16^ ^]^ In addition, the rapid dissolution of CaCO_3_ in the coral skeleton limits the clinical use of coral scaffolds.^[^ ^14^ ^]^ In addition, given the importance of protecting coral reefs,^[^ ^17^, ^18^ ^]^ directly utilizing coral skeletons in regenerative biomedicine may not be environmentally sustainable.

The Chinese proverb “Give a man a fish, or teach a man to fish” reflects the option to either provide immediate assistance or empower with knowledge. This concept underlies our investigation of the mechanisms by which coral proteins promote calcification. The aim is to utilize their potential to promote bone formation within biomaterials. The organic proteins within coral skeletons exhibit a structured and diverse organization that aligns with the diel calcification rhythm.^[^ ^19^ ^]^ These proteins may help control the deposition and stabilization of calcium carbonate, thereby contributing to the overall framework and strength of the coral skeleton. Galaxin is a secretory skeletal matrix protein identified in *Galaxea fascicularis*.^[^ ^20^ ^]^ In conjunction with other cysteine‐rich skeletal organic matrix proteins (SOMPs), it influences the external structure and regulation of crystallization.^[^ ^21^ ^]^ Galaxin is expressed significantly during the post‐settlement stages of coral development. This indicates its involvement in the formation of the organic matrix framework and skeleton.^[^ ^21^ ^]^ These observations imply the significant osteogenic potential of galaxin and thereby, its role in biomaterial applications for bone regeneration. However, the precise role of galaxin in the regulation of calcium deposition in corals remains ambiguous. Nonetheless, its potential regulatory function in coral calcium deposition provides a novel perspective for understanding biomineralization mechanisms and could have significant implications for biomedical engineering. Acknowledging the potential of this protein, we investigated its capability to induce bone regeneration. Rather than directly using the skeletal composition of corals, we drew inspiration from the intricate mechanisms of coral skeleton formation. This approach enabled us to evaluate the potential of galaxin. Based on prior research, we postulated that recombinant galaxin could significantly promote osteogenesis in mouse pre‐osteoblasts and mandibular bone defects.

In this study, we evaluated the osteogenic potential of recombinant galaxin as a novel bioinspired alternative. Our observations demonstrated that recombinant galaxin exerts potent osteogenic effects both in vitro and in vivo. Specifically, recombinant galaxin binds to ATP synthase subunit β (ATP5B) in mouse pre‐osteoblasts. This enhances the ATP production, mitochondrial oxygen consumption rate (OCR), and mitochondrial membrane potential (ΔΨm), which collectively promote osteogenesis. Additionally, this enhancement in mitochondrial activity coincides with a reduction in the reactive oxygen species (ROS) and mitochondrial superoxide (MitoSOX) levels. This indicates a protective effect against oxidative stress. To assess its therapeutic potential, we developed a galaxin‐conjugated gelatin methacryloyl (GelMA) composite and implanted it into mandibular defects in mice. The composite exhibited potential bone‐regeneration capabilities in vivo, which further supports the use of recombinant galaxin in bone tissue engineering. These results emphasize the potential of the recombinant galaxin protein as a novel bioinspired alternative to conventional coral scaffolds. This, in turn, expands the scope of applications in the field of biomedical regeneration.

## 2 Results and Discussion

### 2.1 Recombinant Galaxin Promotes Osteogenesis of Mouse MC3T3‐E1 Pre‐Osteoblasts

**Figure 1:**
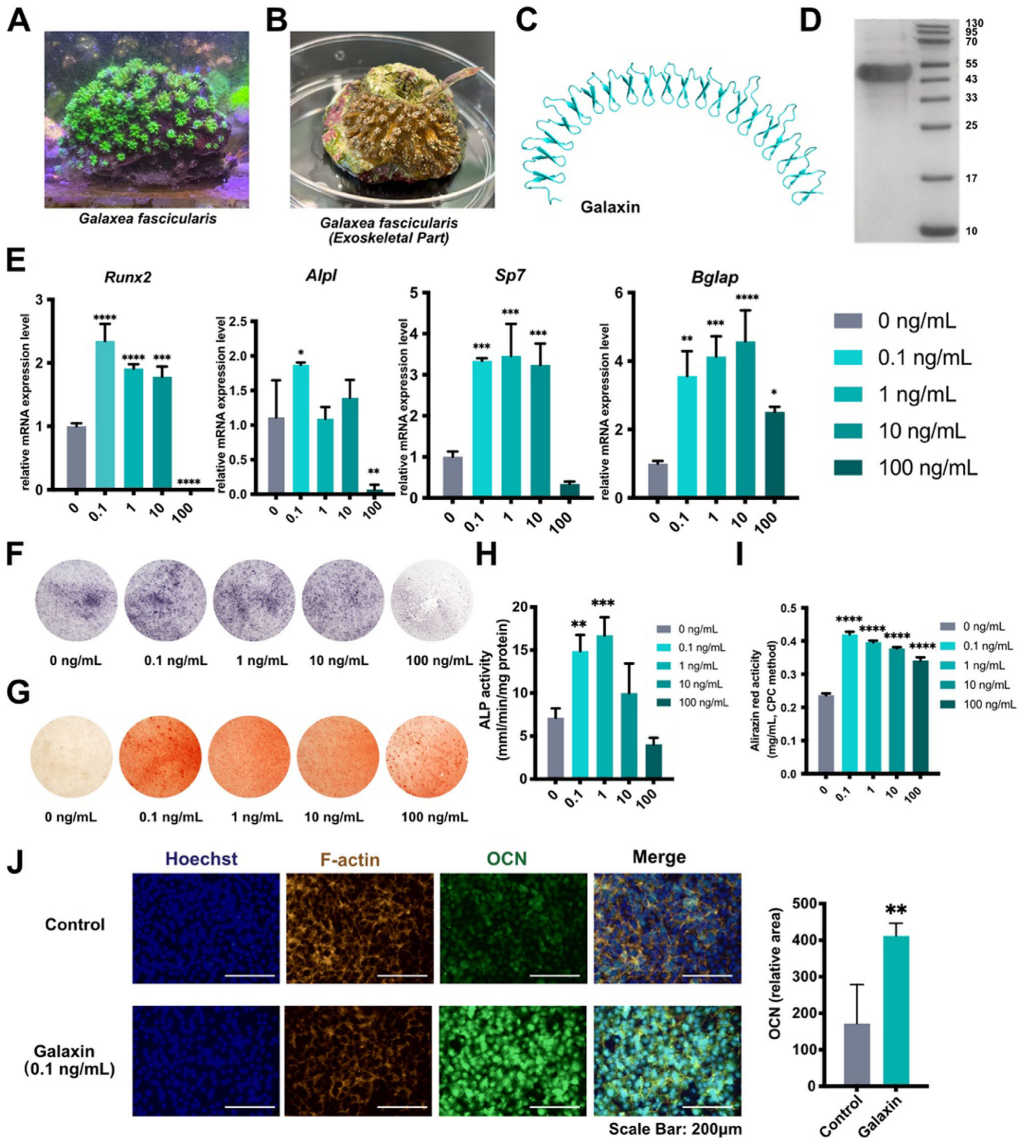
Recombinant galaxin is expressed endogenously and promotes osteogenesis of MC3T3‐E1 pre‐osteoblasts.

The galaxin was obtained from the exoskeletal part of *Galaxea fascicularis* (**Figure** **1**A,B). The nucleotide sequence of cDNA sequence for galaxin was based on Fukuda et al. (GenBank/EMBL/DDBJ Accession No.: AB086183),^[^ ^20^ ^]^ and its protein structure was predicted using AlphaFold3 (Figure 1C). Recombinant galaxin combined with a His‐tag was generated in the HEK293 cell system and subsequently purified. A Sodium Dodecyl Sulfate‐polyacrylamide Gel Electrophoresis (SDS‐PAGE) analysis revealed that recombinant galaxin migrated to 43–55 kDa (Figure 1D). This is consistent with the observations reported by Fukuda et al.^[^ ^20^ ^]^ The purity of galaxin was verified using high‐performance liquid chromatography (HPLC; Figure S1A and Table S1, Supporting Information). The protein stability was determined using the bicinchoninic acid (BCA) method (Figure S1B, Supporting Information). In the evaluation of the biosafety in vitro, the Cell Counting Kit‐8 (CCK‐8) results revealed no significant effects on days 1 and 4. On day 7, the 0.1 and 1 ng mL^−1^ groups showed a marginal increase in cell proliferation. Meanwhile, no significant decrease was observed in the other groups (Figure S1C, Supporting Information). This indicated the biosafety of galaxin in vitro. The quantitative real‐time polymerase chain reaction (qRT‐PCR) results showed that the expression levels of osteogenic genes (*Runx2, Alpl, Sp7*, and *Bglap*) treated with 0.1 ng mL^−1^ galaxin were significantly higher than those in the control group (Figure 1E). With the increase in galaxin concentrations, the expression levels of osteogenic genes exhibited a bell‐shaped curve (Figure 1E). Consistently, qualitative assays demonstrated that 0.1 or 1 ng mL^−1^ galaxin produced the highest levels of alkaline phosphatase (ALP) activity and Alizarin Red staining among the experimental groups (Figure 1F–I). Furthermore, the immunofluorescence staining results showed that the OCN level in the 0.1 ng mL^−1^ galaxin group was significantly higher than that in the control group (Figure 1J). These results indicated that recombinant galaxin effectively promoted osteogenesis in pre‐osteoblasts.

A) *Galaxea fascicularis* in an aquarium. B) The exoskeletal organic part of *Galaxea fascicularis* can be observed when it is out of water. C) Predicted structure of galaxin. D) His‐tag fusion constructs were transfected into HEK293 cells, and His‐tag fusion protein was detected by western blot analysis. E) QRT‐PCR analysis of osteogenesis‐related genes expression in MC3T3‐E1 cells incubated with different concentrations of galaxin (n = 3). F) Representative ALP staining of MC3T3‐E1 cells treated with different concentrations of galaxin. G) Representative alizarin red staining of MC3T3‐E1 cells treated with different concentrations of galaxin. H) Quantification of ALP staining using para‐nitrophenyl phosphate (pNPP) method (n = 3). I) Quantification of alizarin red staining using cetylpyridinium chloride (CPC) method (n = 3). J) Immunofluorescence images of OCN expression and quantification analysis of MC3T3‐E1 cells treated with or without 0.1 ng mL^−1^ galaxin (n = 5). The bars indicate mean ± standard error of the mean. Multiple comparisons were performed using a one‐way ANOVA followed by Tukey's post‐hoc test. Student's *t*‐test was used to compare the means of the two experimental groups. ^*^ *P* < 0.05, ^**^ *P* < 0.01, ^***^ *P* < 0.001, and ^****^ *P* < 0.0001 comparisons between control and galaxin groups.

Coral calcification is a remarkable feature of biomineralization. Herein, corals construct supportive skeletons from aragonitic CaCO_3_.^[^ ^4^ ^]^ The scleractinian coral *Galaxea fascicularis* lives in shallow tropical areas. Its skeleton is composed of many minute calcium skeletal units called polyps.^[^ ^22^ ^]^ As the polyps grow, these secrete calcium and gradually form hard skeletons. The organic proteins within coral skeletons exhibit highly ordered heterologous structures consistent with a diel calcification pattern.^[^ ^19^ ^]^ Among these proteins, galaxin is a secretory skeletal matrix protein identified in *Galaxea fascicularis*.^[^ ^20^ ^]^ It contains tandem repeat motifs containing di‐cysteine sequences (cys‐cys). A previous study reported that galaxin and other cys‐rich skeletal organic matrix proteins (SOMPs) may be involved in the extracellular framework and control of crystallization.^[^ ^21^ ^]^ This highlights the osteogenic role of galaxin.

However, whether and how galaxin controls calcium deposition in corals has not been elucidated. Meanwhile, the potential role of galaxin in regulating coral calcium deposition presents a novel perspective for understanding the biomineralization mechanisms, which could have significant implications for biomedical engineering. To investigate this further, we expressed recombinant galaxin and examined its effects on mouse pre‐osteoblast cell lines. We observed that a low concentration (0.1 ng mL^−1^) of galaxin was sufficient to promote osteogenesis. This highlighted the potential therapeutic value of galaxin in mammalian regenerative medicine.

Coral scaffolds have been used in bone tissue engineering because of their inherent porosity, weight‐bearing capacity, and favorable degradation profiles.^[^ ^7^, ^8^ ^]^ However, the effectiveness of coral scaffolds in promoting bone regeneration relies on the identification of the optimal combination of coral species with optimal mechanical strength and cellular compatibility.^[^ ^16^ ^]^ Previous studies have shown that coral scaffolds exhibit limited osteogenic induction compared with other materials^[^ ^6^ ^]^ such as collagen‐coated PLLA nanofibers.^[^ ^23^ ^]^ By utilizing the recombinant galaxin protein, we succeeded in broadening the applications of bioinspired tissue engineering in functional bioactive scaffolds^[^ ^24^ ^]^ and potentially improving treatment outcomes. This approach contributes to the preservation of coral reef ecosystems by mitigating habitat degradation and maintaining biodiversity.

### 2.2 Recombinant Galaxin Regulates ATPase Activity and Forkhead Box O (FOXO) Pathway of Mouse Pre‐Osteoblasts

**Figure 2:**
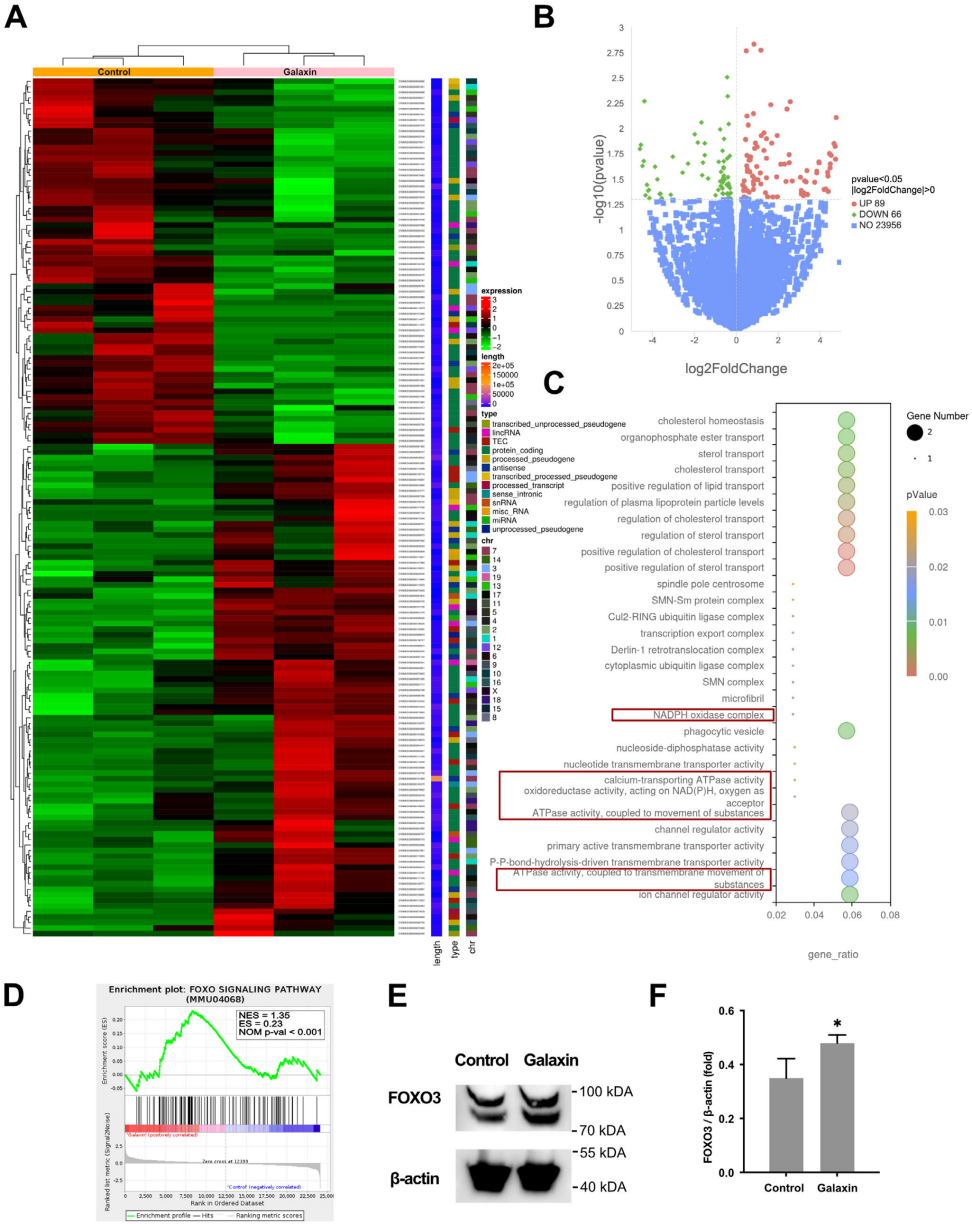
Galaxin modulates ATPase activity and Forkhead Box O3 (FOXO3) signaling pathway.

To further investigate the effects of recombinant galaxin, RNA‐seq was performed on preosteoblasts treated with or without galaxin (**Figure** **2**A). A total of 89 genes were upregulated, whereas 66 genes were downregulated in the galaxin‐treated group (Figure 2B). A gene ontology (GO) enrichment analysis of the upregulated genes revealed that several GO terms were related to the ATPase activity and NADPH oxidase complex (Figure 2C). A gene set enrichment analysis (GSEA) indicated a significant regulation of the Forkhead Box O (FOXO) signaling pathway in the galaxin‐treated group compared with that in the control group (Figure 2D). A western blot analysis showed that galaxin simulated the expression of FOXO3 in pre‐osteoblasts (Figure 2E,F).

Our RNA‐seq results demonstrate that galaxin may affect the ATPase activity and FOXO signaling pathway in pre‐osteoblasts. ATP has been reported to be a source of phosphate and effectively promotes mineral deposition in pre‐osteoblast cell cultures.^[^ ^25^ ^]^ The ATPase activity also plays a significant role in mineralization.^[^ ^25^, ^26^, ^27^ ^]^ The AMPK‐FOXO3a axis in mammals is crucial for maintaining the balance between energy‐producing and energy‐consuming pathways.^[^ ^28^, ^29^, ^30^ ^]^ FOXO decreases oxidative stress, thereby positively regulating bone formation.^[^ ^31^ ^]^ FOXO3 knockdown has been shown to induce high ROS levels, thereby regulating the osteogenic differentiation of human mesenchymal stem cells (MSCs).^[^ ^32^ ^]^ In a study on aged mice, the overexpression of FOXO3 demonstrated its potential to prevent bone loss by downregulating the activity of PPAR‐γ and the Notch signaling pathways.^[^ ^33^ ^]^ Consistent with these reports, our results revealed that galaxin increases the ATPase activity and FOXO3 signaling pathway, and may ultimately promote osteogenesis by regulating the energy metabolism.

A) Heatmap representation of differentially expressed genes in RNA‐seq data from galaxin‐treated group versus that from control group in MC3T3‐E1 cells (n = 3). B) Volcano plot of the different expressed genes from galaxin‐treated group versus that from control group. C) Gene ontology (GO) enrichment analysis of differentially expressed genes. D) Gene set enrichment analysis (GSEA) for FOXO signaling pathway. E) Representative western blotting analysis of FOXO3 and β‐actin after treatment with or without galaxin. F) Quantification of western blotting results as FOXO3/ β‐actin. The bars indicate mean ± standard error of the mean (n = 3). Student's *t*‐test was used to compare the means of the two experimental groups. ^*^ *P* < 0.05.

### 2.3 Recombinant Galaxin Interacts with ATP5B and Regulates Mouse Pre‐Osteoblasts Through Mitochondrial Metabolism

**Figure 3:**
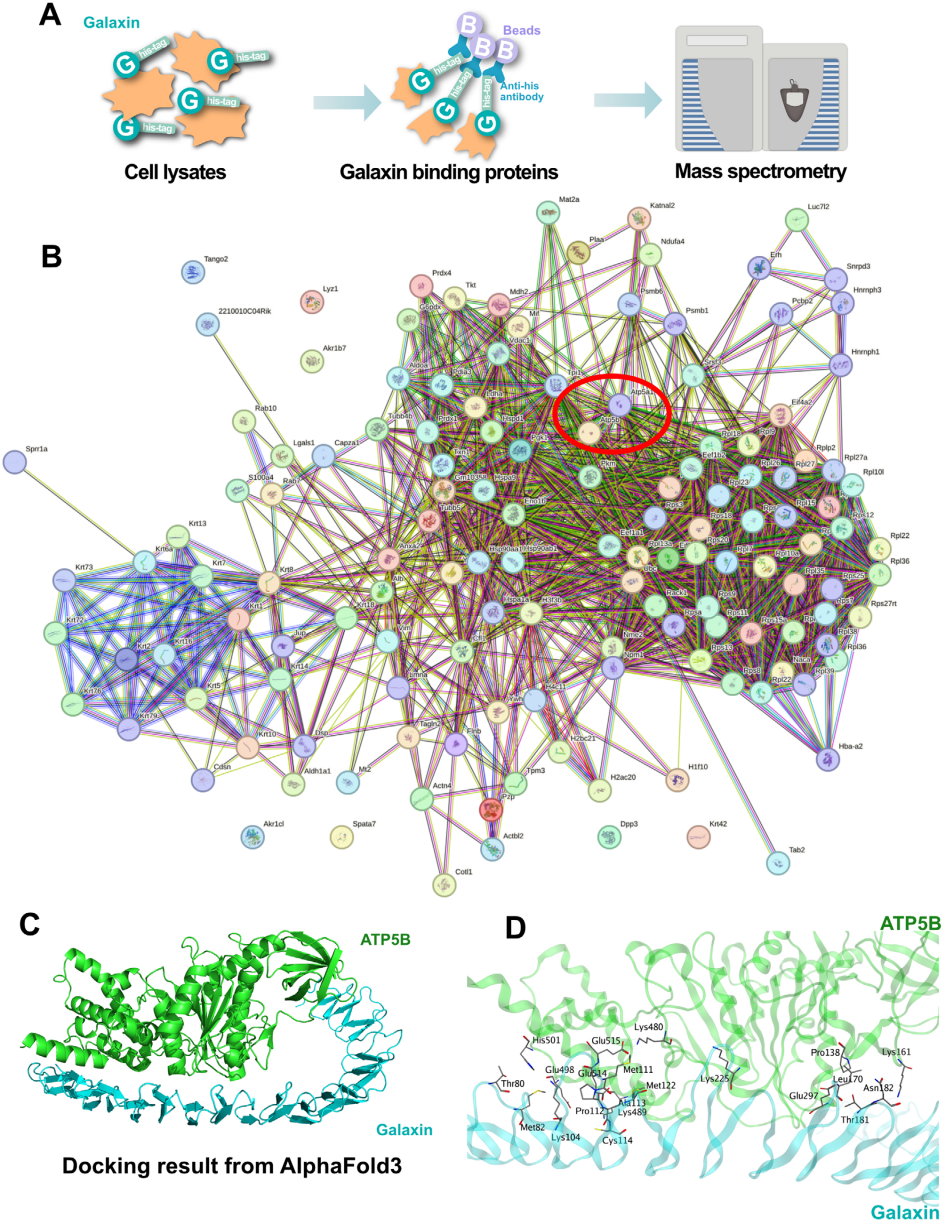
Galaxin targets ATP5B in MC3T3‐E1 Pre‐osteoblasts.

To identify the potential molecules that interact with galaxin, we performed immunoprecipitation experiments followed by mass spectrometry (**Figure** **3**A). The hub proteins were identified via STRING online software, including ATP5a1 and ATP5b (Figure 3B). ATP5B is a major component of the catalytic center of the ATP synthase complex, which is closely related to the mitochondrial function and is responsible for the generation of ATP.^[^ ^34^, ^35^, ^36^ ^]^ Next, we predicted the binding potential of galaxin to ATP5B through Molecular Operating Environment (MOE) (Figure S2, Supporting Information)^[^ ^37^ ^]^ and AlphaFold3 (Figure 3C).^[^ ^38^ ^]^ The binding mode between galaxin and ATP5B was determined using MOE (Figure 3D and Table S2, Supporting Information). Additionally, confocal microscopy experiments using His‐tagged galaxin revealed its intracellular localization (Figure S3A, Supporting Information). Transmission electron microscopy (TEM) indicated the presence of vesicles within the cells (Figure S3B, Supporting Information), thereby implying that galaxin may enter the cell through endocytosis.

ATP synthase is also known as F_1_F_0_‐ATPase or complex V. It is responsible for generating the majority of cellular ATP and consists of F_0_ and F_1_. The F1 domain consists of α, β, γ, δ, and ε subunits. These are encoded by ATP5A, ATP5B, ATP5C, ATP5D, and ATP5E, respectively.^[^ ^39^, ^40^ ^]^ Molecular docking analyses revealed an interaction between galaxin and ATP5B. Consistent with the previous RNA‐seq results, the mass spectrometry results also showed a correlation between galaxin and energy metabolism. The upregulation of ATPase pathway genes observed in the RNA‐seq analysis emphasizes the crucial role of mitochondrial energy production in this biological process. This increased ATPase activity indicates an increased demand for ATP synthesis. This implies that galaxin‐treated MC3T3‐E1 cells actively engaged in energy‐intensive processes, likely driven by an enhanced mitochondrial activity.

A) Cell lysates were incubated with his‐Galaxin. Then, galaxin binding proteins were pulled down using antibodies and beads, and these were analyzed via mass spectrometry. B) The galaxin binding proteins were mapped, and hub proteins were identified. C) The docking results of galaxin and ATP5B from AlphaFold3. D) The docking of galaxin and ATP5B highlighting the amino acids involved in the interaction.

**Figure 4:**
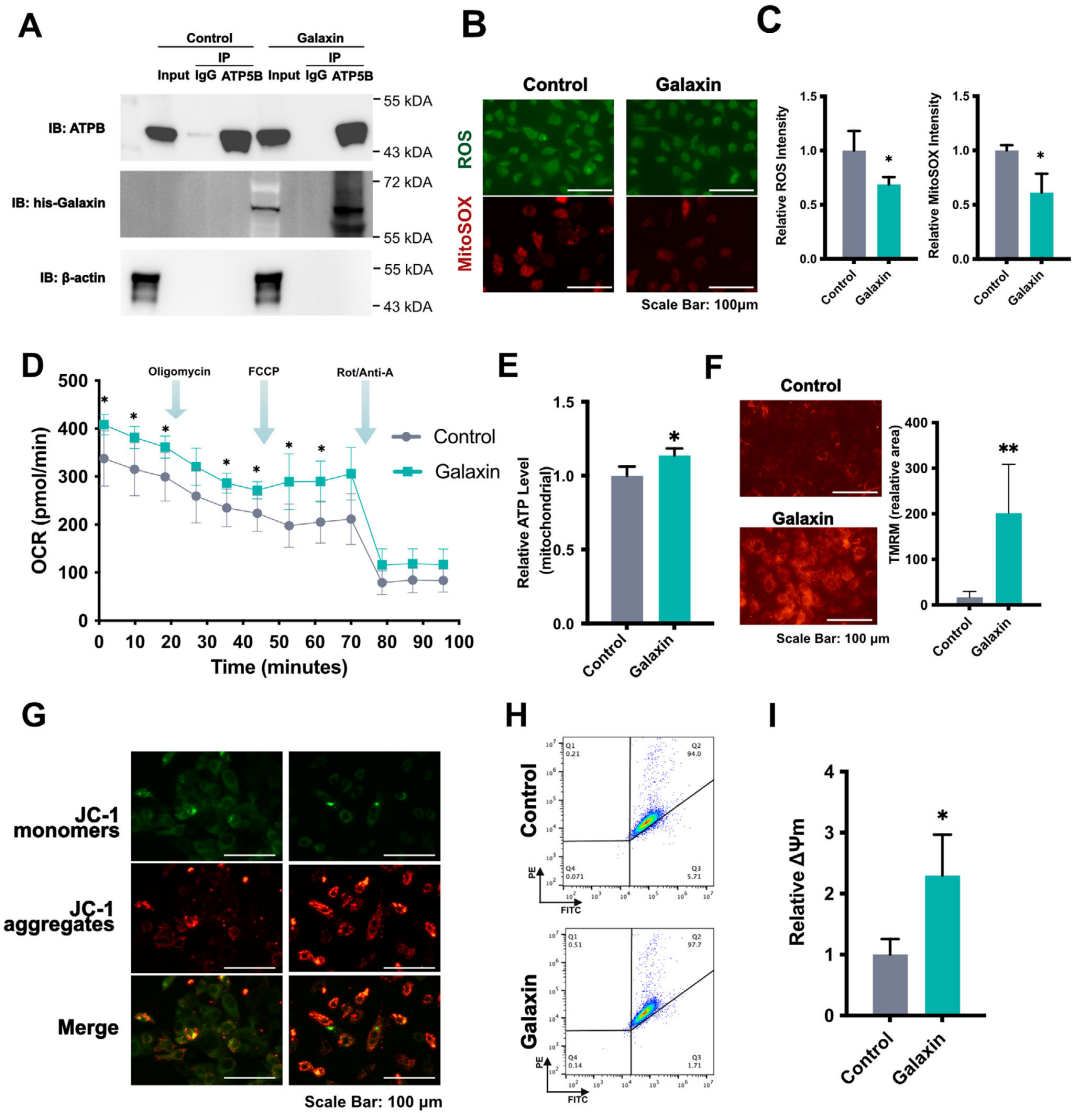
Galaxin is related to mitochondrial metabolism and ATP production.

Immunoprecipitation assays verified that galaxin formed a complex with ATP5B (**Figure** **4**A). No bands corresponding to His‐tagged galaxin were detected in the control group after immunoprecipitation with the ATP5B antibody. However, both input and immunoprecipitated samples from the galaxin‐treated group displayed bands between 55 and 72 kDa. This exceeded the anticipated molecular weight of galaxin (53 kDa). This indicates that galaxin may undergo glycosylation in cells (Figure 4A). To detect the mitochondrial and intracellular ROS levels, immunofluorescence staining was performed (Figure 4B). The results revealed a significant reduction in both intracellular and mitochondrial ROS levels in pre‐osteoblasts cultured with galaxin compared with the control group (Figure 4B,C). The mitochondrial stress test revealed that galaxin‐treated cells had a higher basal oxygen consumption rate (OCR) than the control cells (Figure 4D). The significant difference in the initial stage indicates that galaxin promotes the overall metabolic activity of the cells. ATP‐linked respiration (after oligomycin was injected) was also significantly higher in the galaxin‐treated group than in the control group (Figure 4D). During this stage, oligomycin was added to the cells to prevent ATP production. This implies that galaxin may affect mitochondrial function. Furthermore, after the injection of carbonyl cyanide 4‐(trifluoromethoxy) phenylhydrazone (FCCP), the OCR of the galaxin‐treated group significantly increased the OCR compared with the control group (Figure 4D). FCCP injection caused a maximal increase in the OCR, which represents the maximum respiratory capacity of the cells. The significant difference observed after FCCP injection further supports the hypothesis that galaxin affects the mitochondrial function related to the electron transportation chain or other processes. However, no significant differences were observed in the non‐mitochondrial respiration (after Rot/Anti‐A injection) between the galaxin and control groups (Figure 4D). The absence of a significant difference between the groups after Rot/Anti‐A injection indicates that galaxin may not directly affect the electron transportation chain. In addition, the galaxin treatment promoted mitochondrial ATP production compared with that in the control group (Figure 4E).

The mitochondrial membrane potential (ΔΨm) was first measured by tetra‐methylrhodamine methyl ester (TMRM) dye. The higher level of TMRM fluorescence was observed in the galaxin‐treated group (Figure 4F). A JC‐1 fluorescent probe was also applied to measure ΔΨm, which transitioned from red aggregates to green monomers as ΔΨm decreased. Green and red fluorescence were observed under a microscope (Figure 4G). Meanwhile, a quantitative analysis was performed using flow cytometry (Figure 4H,I). We observed that galaxin significantly promoted the relative ΔΨm compared with the control group (Figure 4I).

Osteoblasts require a significant amount of energy during the synthetic phase of differentiation.^[^ ^41^ ^]^ Moreover, the mitochondrial function plays a positive role in the osteogenic differentiation of osteoblasts,^[^ ^42^ ^]^ ligament cells,^[^ ^43^ ^]^ and mesenchymal stem cells.^[^ ^44^, ^45^, ^46^ ^]^ Our results revealed that galaxin also regulates mitochondrial function. By interacting with ATP5B, galaxin generates a higher basal oxygen consumption rate, ATP‐linked respiration, and ATP production. Decreased intracellular and mitochondrial ROS levels also imply a protective role for galaxin against oxidative injury. Although excessive ROS can be detrimental, tightly controlled ROS levels function as signaling molecules that influence osteoblast differentiation and bone mineralization.^[^ ^47^, ^48^ ^]^ Furthermore, the present study observed that galaxin upregulated the ΔΨm of pre‐osteoblasts. Similarly, an increased ΔΨm has been also observed in magnesium‐iron‐induced osteogenesis of bone marrow stem cells,^[^ ^49^ ^]^ and a decrease in ΔΨm has been observed when osteogenic differentiation is suppressed and mitochondrial energy metabolism is impacted.^[^ ^50^ ^]^ ΔΨm is directly related to the ATP production through oxidative phosphorylation within mitochondria. Thus, it regulates the metabolic and osteogenic regulation in MSCs.^[^ ^51^ ^]^ In addition, mitochondria may control the gene ratio of ROS, thereby influencing the osteogenic differentiation of MSCs.^[^ ^52^ ^]^ Polyphenol‐cysteine nanoparticles have been assembled to scavenge mitochondrial ROS and maintain the mitochondrial homeostasis. Thereby, these have provided therapeutic insights into bone regeneration.^[^ ^53^ ^]^

Investigating the relationship between mitochondrial metabolism and osteogenesis broadens the therapeutic potential of targeting mitochondrial functions. Recombinant galaxin enhances the mitochondrial function, thereby providing a potential strategy for enhancing bone regeneration in clinical situations. This makes it a potential candidate for future bone regenerative therapies.

A) Co‐IP assays showing the interaction between galaxin and ATP5B in MC3T3‐E1 pre‐osteoblasts. B) The mitochondrial and intracellular reactive oxygen species (ROS) were measured using MitoSOX and DCFH‐DA staining, respectively. C) Quantification of mitochondrial ROS and intracellular ROS (n = 3). D) The oxygen consumption rate (OCR) profile was detected in MC3T3‐E1 cells with a Seahorse XF24 analyzer. The metabolic inhibitors were injected at different time points as indicated (n = 3). E) Relative ATP level of mitochondria with galaxin treatment (n = 3). F) Representative fluorescent images and quantification of tetra‐methylrhodamine methyl ester (TMRM) dye with galaxin treatment (n = 5). G) The mitochondrial membrane potential (MMP) was detected using the fluorescent probe, JC‐1. JC‐1 monomers, green; JC‐1 aggregates, red. H) Representative flow cytometry images of MC3T3‐E1 cells after incubation with JC‐1 probe. I) The relative red/green ratio (relative ΔΨm) of cells stained by JC‐1 probe. The bars indicate mean ± standard error of the mean (n = 3). Student's *t*‐test was used to compare the means of the two experimental groups. ^*^ *P* < 0.05, ^**^ *P* < 0.01.

### 2.4 ATP Synthesis Inhibitor Oligomycin Impairs Osteogenic Effects Of Galaxin

**Figure 5:**
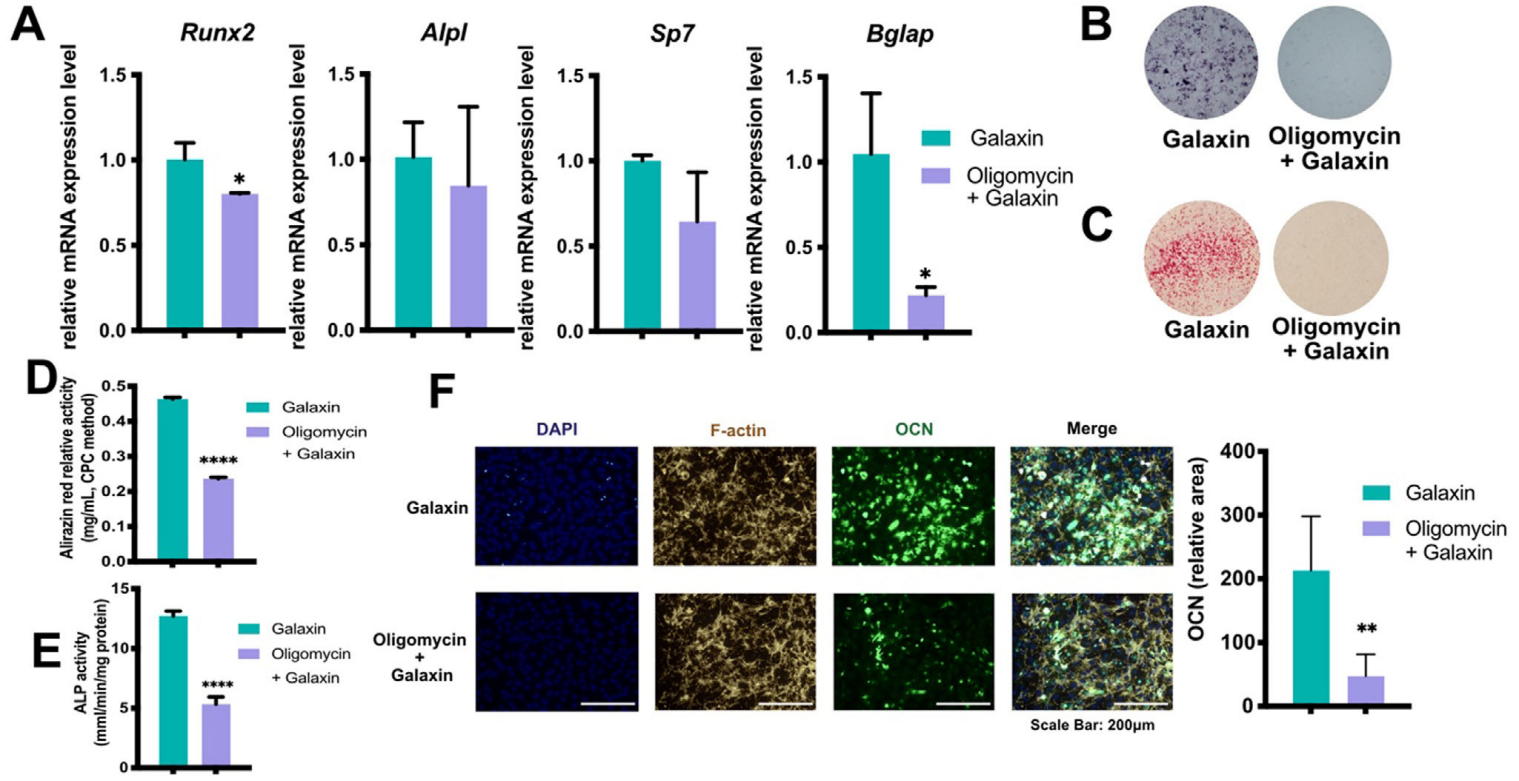
Galaxin requires ATP synthase activity to regulates osteogenesis of pre‐osteoblasts.

To further verify that ATP synthesis is responsible for the osteogenic effects of galaxin, ATP synthesis inhibitor experiments were performed. The osteogenic effect of galaxin on pre‐osteoblasts was neutralized by the ATP synthase inhibitor oligomycin (**Figure** **5**A–F). The qRT‐PCR results showed that the ATP synthase inhibition (oligomycin + galaxin group) significantly decreased the expression of runx2 and bglap in pre‐osteoblasts compared with the galaxin group (Figure 5A). ALP and alizarin red staining assays demonstrated that ATP synthase inhibition also obstructed the osteogenesis of cells (Figure 5B–E). Immunofluorescence staining showed that the OCN levels in the oligomycin group were significantly lower than those in the galaxin group (Figure 5F). These observations show that galaxin promotes the osteogenesis of pre‐osteoblasts in an ATP synthase‐dependent manner.

A) QRT‐PCR analysis of osteogenesis‐related gene expression in MC3T3‐E1 cells incubated with ATP synthase inhibitor oligomycin (1 µm) and with galaxin (0.1 ng mL^−1^) subsequently (n = 3). B) Representative ALP staining of MC3T3‐E1 cells. C) Representative alizarin red staining of MC3T3‐E1 cells. D) Quantification of ALP staining using para‐nitrophenyl phosphate (pNPP) method (n = 3). E) Quantification of alizarin red staining using cetylpyridinium chloride (CPC) method (n = 3). F) Immunofluorescence images and quantification analysis of OCN expression in MC3T3‐E1 cells (n = 5). The bars indicate mean ± standard error of the mean. Student's *t*‐test was used to compare the means of the two experimental groups.^*^ *P* < 0.05, ^**^ *P* < 0.01， ****P < 0.0001.

### 2.5 Recombinant Galaxin Promotes Mandibular Bone Regeneration

**Figure 6:**
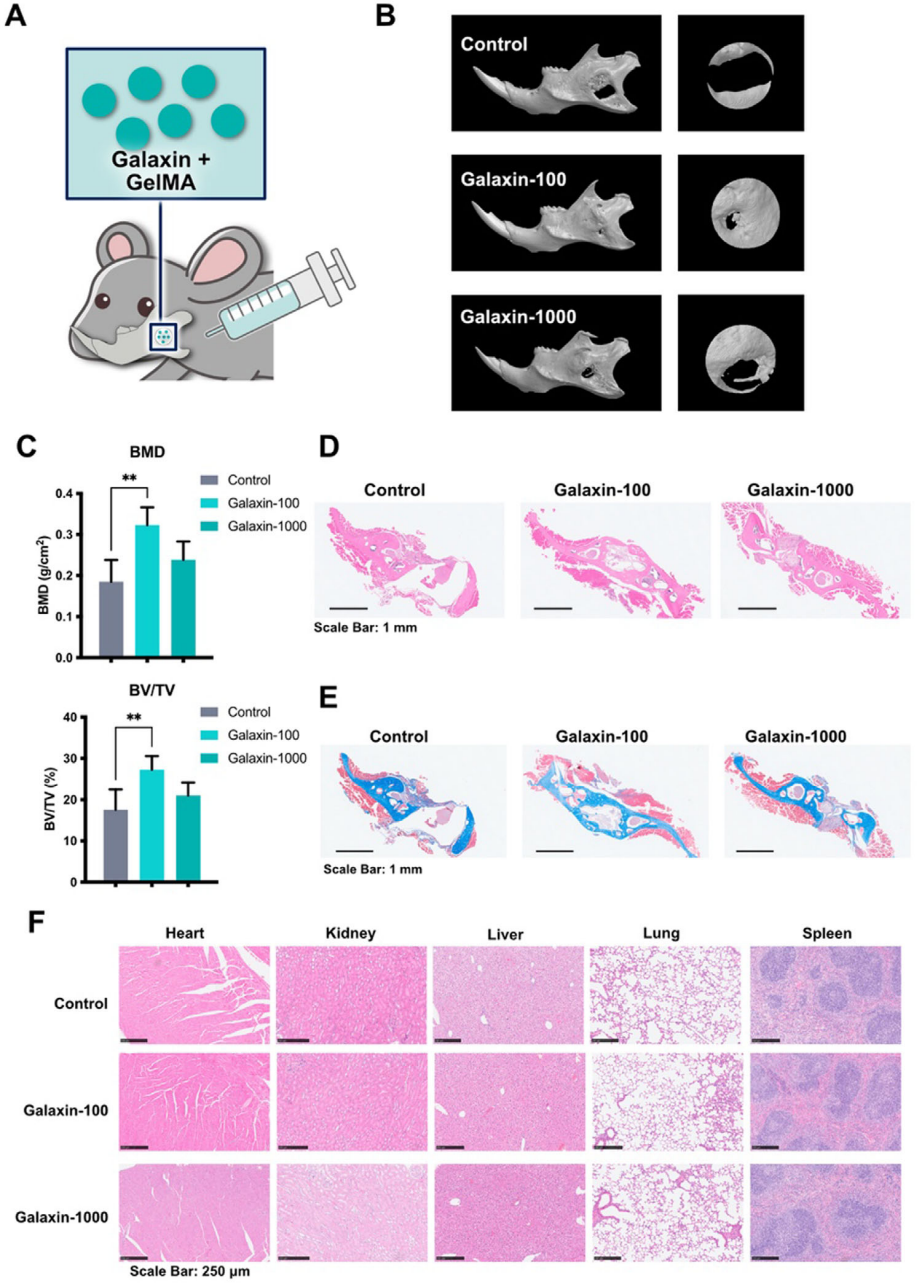
Galaxin promotes osteogenesis in mouse mandibles after mandibular defects.

To investigate the osteogenic effects of galaxin in vivo, mice with penetrating mandibular defects were implanted with galaxin dispersed in gelatin methacryloyl (GelMA) (**Figure** **6**A). Recombinant galaxin was dispersed in 10% GelMA at concentrations of 0 ng mL^−1^ (control group), 100 ng mL^−1^ (Galaxin‐100 group), and 1000 ng mL^−1^ (Galaxin‐1000 group) to form injectable Galaxin‐GelMA composites. Eight weeks post implantation, the morphology of the newly formed bone was examined using micro‐CT (Figure 6B). The Galaxin‐100 group exhibited the best osteogenic potential, with a significantly higher bone mineral density (BMD) and bone volume/total volume ratio (BV/TV) than the control and galaxin‐1000 groups (Figure 6C). Although the Galaxin‐1000 group tended to be superior in increasing the BMD and BV/TV compared with the control group, the differences were not statistically significant (Figure 6C). Hematoxylin and eosin (H&E) and Masson staining further supported the micro‐CT observations. These revealed a large amount of new bone tissue in the defect location of the Galaxin‐100 group, whereas the Galaxin‐1000 group showed less new bone tissue (Figure 6D,E). Furthermore, no significant tissue lesions were observed in the heart, kidney, liver, lungs, or spleen (Figure 6F). This indicated the cytocompatibility of galaxin.

A) Mandibular defects were generated in C57BL/6 mice. Then, different concentrations of galaxin were mixed with GelMA and injected into mandibular defects (n = 6 in each group). B) Representative micro‐CT analysis of bone formation in mandibles of mice implanted with Galaxin‐GelMA after mandibular defects. C) Quantifications of trabecular bone mineral density (BMD) and bone volume/tissue volume ratio (BV/TV) based on micro‐CT analysis. D) Representative H&E staining of mandibular sections. E) Representative Masson staining of mandibular sections. F) Representative H&E staining of heart, kidney, liver, lung sections. The bars indicate mean ± standard error of the mean. Multiple comparisons were performed using a one‐way ANOVA followed by Tukey's post‐hoc test. ^**^ *P* < 0.01.

The combination of galaxin and GelMA hydrogels can potentially be used as a bio‐inspired protein for bone tissue regeneration. Owing to the injectable properties, biocompatibility, and close resemblance of matrix metalloproteinase target sequences to the native extracellular matrix, the incorporation of various biomaterials into GelMA is an effective strategy for tissue engineering.^[^ ^54^, ^55^, ^56^, ^57^ ^]^

Pure GelMA has limited osteogenic potency,^[^ ^58^, ^59^ ^]^ thereby minimizing the risk of confounding factors in evaluating the specific effects of galaxin on bone regeneration. Furthermore, it allows for a clearer assessment of the osteogenic potential of galaxin without interference from the scaffold material. In addition, the injectable characteristic of GelMA allows for minimally invasive administration.^[^ ^60^ ^]^ This facilitates the localized delivery of galaxin directly to the target site. This combination provides a safe, effective, and controlled delivery system for evaluating the osteogenic potential of galaxin. The galaxin‐GelMA composites exhibited the osteogenic potency of galaxin in our study. In our study, coral bio‐inspired recombinant galaxin served as a regulator of ATP synthase. Thereby, it enhanced the mitochondrial function and, ultimately, promoted osteogenesis. The capability of galaxin to promote mitochondrial metabolism and energy production likely contributes to enhanced bone regeneration in preclinical models. Future studies utilizing nuclear magnetic resonance to elucidate the interaction between galaxin and ATP5B in pre‐osteoblasts or other cell types would provide effective insights into the precise design of recombinant proteins or peptides for bone regeneration biomaterials with metabolic functions. To translate galaxin into clinical applications, further in‐depth long‐term safety and dose optimization studies are required to ensure the safety and efficacy of galaxin in human bone regeneration. In addition, more effective and convenient delivery methods, the potential of combining galaxin with other osteoinductive factors, and its application to different types of bone defects should be studied.

## 3 Conclusion

Recombinant galaxin plays an osteogenic role in vitro and in vivo. Mechanistically, recombinant galaxin enhanced the mitochondrial function of preosteoblasts and protected these from oxidative stress. Recombinant galaxin was characterized as a regulator of ATP synthase to enhance OCR, ATP production, and ΔΨm. In addition, the ROS and MitoSOX levels decreased following galaxin treatment. Our observations provide insights into the study of bioinspired coral proteins and highlight novel biomimetic proteins for application in bone tissue engineering.

## 4 Experimental Section

### Cell Culture

MC3T3‐E1 pre‐osteoblasts cells were cultured in α‐MEM medium (Gibco) supplemented with 10% fetal bovine serum (Biological Industries) and 100 U mL^−1^ penicillin‐streptomycin (HyClone, Thermo Scientific). The cells were cultured in humidified air with 5% CO_2_ at 37 °C.

### Generation of Recombinant Galaxin and Verification of Effect on MC3T3‐E1 Cells

Recombinant galaxin combined with His‐tag was generated in the HEK293 cell system and purified.^[^ ^61^ ^]^ (NB Bio Lab) The His‐Galaxin was assessed by western blotting analysis with anti‐His antibody (Yeasen Biotechnology). The purity of galaxin was verified by HPLC at a flow rate of 0.7 mL mi^−1^n and wavelength of 280 nm. A CCK‐8 assay was performed using a CCK‐8 kit (Bioground Biotech), and the optical density (OD) was measured at 450 nm. The effect of recombinant galaxin on MC3T3‐E1 cells were verified by qRT‐PCR, ALP staining, alizarin red staining, and immunofluorescence analysis. qRT‐PCR was performed on a LightCycler 96 (Roche) using SYBR Green Mix (Takara). A gene expression analysis was performed using the ΔΔCt method with GAPDH normalization. Cells were fixed with 4% paraformaldehyde solution and stained with the BCIP/NBT ALP Color Development Kit (Beyotime) or 1% alizarin red staining (pH 4.2, Solarbio) for qualitative testing. ALP was quantified using the para‐nitrophenyl phosphate (pNPP) method (Beyotime) to detect the OD value at 405 nm. Alizarin red was quantified using the cetylpyridinium chloride (CPC) method (Solarbio), and the OD value was measured at 560 nm. For immunofluorescence staining, after being treated with galaxin for four days, MC3T3‐E1 cells were permeabilized with 0.1% Triton X‐100, fixed with 4% paraformaldehyde solution, and blocked with 5% bovine serum albumin (BSA). Following overnight incubation, primary anti‐OCN Abs (Yeasen Biotechnology), FITC Goat Anti‐Rabbit IgG (Yeasen Biotechnology), and F‐actin (Yeasen Biotechnology) were used to facilitate nuclear staining. For the intracellular localization analysis, a His‐tag antibody (Yeasen Biotechnology) was used, followed by FITC goat anti‐rabbit IgG. Leica fluorescence and confocal microscopy images were obtained by immunofluorescence staining. The immunofluorescence staining of OCN was quantified using ImageJ software (NIH).

### Transcriptome Sequencing Analysis

After treatment with galaxin for one day, the total RNAs was extracted from the MC3T3‐E1 cells. RNA sequencing and associated analyses were performed using Novogene (Beijing, China). DESeq 2 was used to analyze differentially expressed genes (DEGs) by employing a *P* value ≤ 0.05 and log2 fold variation ≥ 0 to identify significant variations in the gene expression. To investigate the functional roles of these genes, a GO enrichment analysis was performed using the cluster Profiler R package (3.8.1). A GSEA was conducted using the GSEA tool developed by Novegene (Beijing, China).

### Western blot analysis

MC3T3‐E1 cells were lysed using RIPA buffer supplemented with protease inhibitors. Protein lysates were separated using 10% sodium dodecyl sulfate‐polyacrylamide gel electrophoresis (SDS‐PAGE) and transferred onto PVDF membranes (Millipore). After blocking with 5% BSA, the membranes were incubated with primary antibodies targeting FOXO3 (ABclonal Technology) and β‐actin (Yeasen Biotechnology). Detection was performed using appropriate secondary antibodies (Cell Signaling Technology). The protein bands were quantified using ImageJ software (NIH), with the FOXO3 levels normalized to β‐actin. For the immunoprecipitation (IP) experiments, the cells were lysed using IP lysis buffer supplemented with protease inhibitors. Then, cells were incubated with ATPB antibody (Abcam) overnight at 4 °C and then incubated with protein A/G magnetic beads (MedChemExpress) for 2 h. Subsequently, the beads were washed and collected through a magnetic stand, and a western blot analysis was performed using primary antibodies targeting ATPB antibody, his‐tagged antibody, and β‐actin.

### Mass Spectrometry Analysis

Cell lysates were incubated with an anti‐His antibody (Yeasen Biotechnology). Protein A/G magnetic beads (MedChem Express) were then used to pull down the galaxin‐antibody complexes in conjunction with the interacting proteins. The isolated protein complexes were analyzed by mass spectrometry. An Orbitrap Exploris 480 mass spectrometer (Thermo Fisher Scientific) coupled with FAIMS technology was used for the analysis. The key settings included a Nanospray Flex ion source (Thermo Scientific), a spray voltage of 2.1 V, and an ion transportation capillary temperature of 320 °C. The hub proteins were identified using STRING online software.

### Molecular Docking

Molecular docking of mouse ATP5B (AlphaFold ID: AF‐P56480‐F1) and *Galaxea fascicularis* galaxin (AlphaFold ID: AF‐Q8I6S1‐F1) was performed by AlphaFold3^[^ ^38^ ^]^ and MOE. The MOE plugin “protein‐protein docking” was used to study the interaction between ATP5B and galaxin. Both the proteins were protonated using the AMBER10:EHT force field. The docking process employed a coarse‐grained representation of the proteins and a fast Fourier transform for efficiently evaluating potential binding modes. The induced‐fit scheme was implemented to refine the coarse‐grained model and optimize the side chains of the residues at the protein‐protein interface. Subsequently, the docking poses were subjected to energy minimization to identify the most favorable binding conformations. The binding energies of the refined poses were calculated using the GB/VI scoring function.

### Measurement of Intracellular and Mitochondrial ROS

This study assessed the ROS levels in different cellular compartments. The intracellular ROS were measured by incubating cells with DCFH‐DA (Beyotime) at 37 °C for 20 min, followed by fluorescence microscopy. The mitochondrial ROS levels were determined using MitoSOX Red probe (Beyotime) and fluorescence imaging. The fluorescence intensity was measured using ImageJ software (NIH).

### Measurement of cell OCR

A Seahorse Bioscience XF‐24 Extracellular Flux Analyzer was used to measure the OCR. Cells were seeded and left overnight in XF24‐well cell culture microplates. At the designated times, several inhibitors (Agilent Technologies) were injected: 1.5 µm oligomycin was added to assess the respiration related to the ATP turnover, 5.0 µm carbonyl cyanide 4‐(trifluoromethoxy) phenylhydrazone (FCCP) was used to induce maximal respiration, and rotenone/antimycin A (Rot/Anti‐A) was added at a final concentration of 0.5 µm to inhibit the electron transportation to detect the non‐mitochondrial basal respiration level.

### ATP Level Measurement

The intracellular ATP levels were determined using an enhanced ATP Assay Kit (Beyotime Biotechnology) according to the manufacturer's instructions. The cells were then treated with galaxin for four days. To measure the mitochondrial ATP levels, cells were incubated with recording buffer containing 5 mm 2‐deoxyglucose (2‐DG, Shanghai Yuanye Bio‐Technology) and 5 mm pyruvate (Biosharp Life Sciences).^[^ ^62^ ^]^ The cell suspensions were centrifuged, and the supernatant was collected for the subsequent analyses. An ATP detection working solution was added to a black 96‐well plate for luminescence analysis using a microplate reader.

### Mitochondrial Membrane Potential (ΔΨm) Measurement

To measure the tetra‐methylrhodamine methyl ester (TMRM), recombinant galaxin‐treated cells were incubated with TMRM (Beyotime Biotechnology) at 37 °C for 20 min and washed with buffer. Images were captured using a fluorescence microscope. Recombinant galaxin‐treated cells were incubated with diluted JC‐1 solution (Beyotime Biotechnology) at 37 °C for 20 min and washed with buffer. Images were captured using a fluorescence microscope. Furthermore, the cells were collected, and the PE and FITC fluorescence values were determined using a flow cytometer (Beckman) according to the manufacturer's instructions.

### Animal Model

Adult male C57BL/6 WT mice were purchased from Gempharmatech (Nanjing, China). The mice were housed in a specific pathogen‐free (SPF) facility with a 12:12 h light/dark cycle. The use of the animals was approved by the Animal Care and Ethics Committee of the West China School of Stomatology, Sichuan University (NO: WCHSIRB‐D‐2024‐711). Mandibular defects were generated as described previously.^[^ ^63^ ^]^ Briefly, after anesthesia, a 5 mm incision was made on the mandibular skin. The masseter muscles were then separated bluntly, and the periosteum was incised. On the mandible, a full‐thickness penetrating defect (2.3 mm diameter) was created using a dental bur that was maintained cold continually by flushing with 0.9% saline. Different concentrations of recombinant galaxins were dispersed in 10% GelMA (EFL‐Tech Co., Ltd.) to form injectable galaxin‐GelMA composites. The galaxin‐GelMA composites were implanted in the penetrating defect area immediately after the operation, and the wound was closed in layers.

### Micro‐CT

Eight weeks after the surgery, the mice were sacrificed. The mandibles were fixed in 4% polymerized formaldehyde. A SkyScan micro‐CT system (Bruker, Billerica, MA, USA) was used to scan the mandible. To prevent dehydration, the samples were maintained in a moist atmosphere. Morphometric analyses were conducted using Bruker CTAn software. The grayscale for 3D reconstruction was established between 120 and 255. The 3D morphometric parameters of the bone microarchitecture (BV/TV and BMD) were computed.

### Histological Analysis

The mandible samples were decalcified for two weeks in PBS with 10% ethylenediaminetetraacetic acid disodium (EDTA·2Na), which was replaced daily. Heart, kidney, liver, lung, and spleen samples were collected and fixed. The samples were dehydrated in gradient ethanol, embedded in paraffin, and sectioned into 5 µm‐thick slices for Hematoxylin and eosin (H&E) and Masson staining. H&E and Masson's trichrome staining were performed according to the standard protocols.

### Statistical Analysis

The data were presented as mean ± standard error of mean and analyzed using Prism 9.3 (GraphPad Software). Multiple comparisons were performed using a one‐way ANOVA followed by Tukey's post‐hoc test. Unpaired Student's t‐test was used to compare the means of the two experimental groups. The statistical significance was set at *P* < 0.05. All the micrograph assays were performed independently at least three times with similar results.

## Conflict of Interest

The authors declare no conflict of interest.

## Supporting information



Supplementary information

## Data Availability

The data that support the findings of this study are available from the corresponding author upon reasonable request.
