# Supplementary material for: Enhancing Osteogenesis through Bio‐Inspired Recombinant Coral Protein Galaxin by Targeting Mitochondrial Metabolism and ATP Production
Source: Adv Sci (Weinh). 2025 Mar 8;12(17):2412867. doi: 10.1002/advs.202412867 (PMC12061279; doi:10.1002/advs.202412867)
Supplement: Supplementary file 1 — Supplementary information [file ADVS-12-2412867-s001.docx]

**Enhancing Osteogenesis through Bio-Inspired Recombinant Coral Protein Galaxin by Targeting Mitochondrial Metabolism and ATP Production**

**Supplementary information**

**Figure S1:**

**
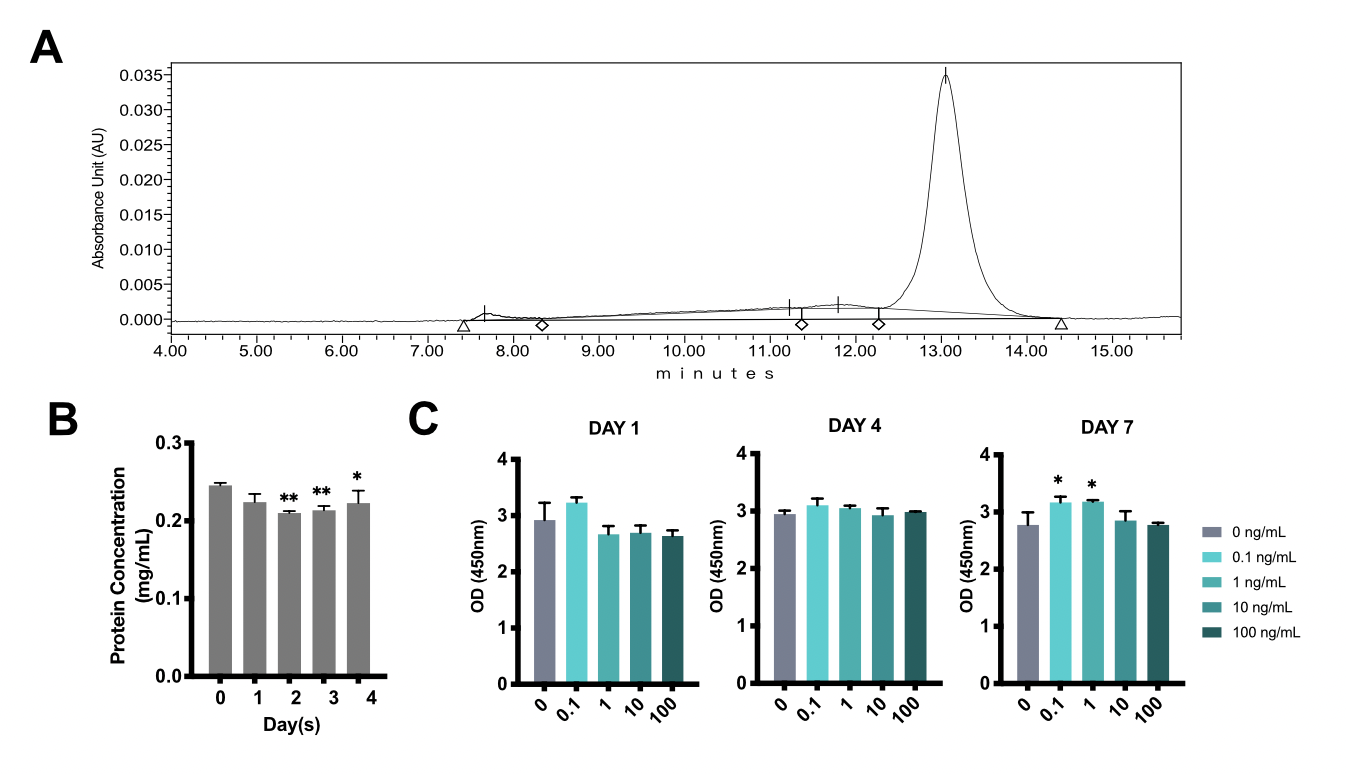
**

Figure S1: (A) High Performance Liquid Chromatography (HPLC) results. (B) The protein concentration of galaxin after 1, 2, 3, 4 days in 4℃ (n = 3). (C) CCK-8 experiments have been applied to MC3T3‐E1 cells after 1, 4, and 7 days (n = 3). Multiple comparisons were performed using a one-way ANOVA followed by Tukey's post-hoc test. * P < 0.05, ** P < 0.01 comparisons between control and galaxin groups.

**Table S1:** **High Performance Liquid Chromatography (HPLC) Results**

| Peak | Duration | % Area |
| --- | --- | --- |
| Peak 1 | 7.661 | 0.014316 |
| Peak 2 | 11.224 | 0.0118 |
| Peak 3 | 11.793 | 0.012212 |
| Peak 4 | 13.049 | 0.961672 |

**Figure S2: Docking result from MOE**

**
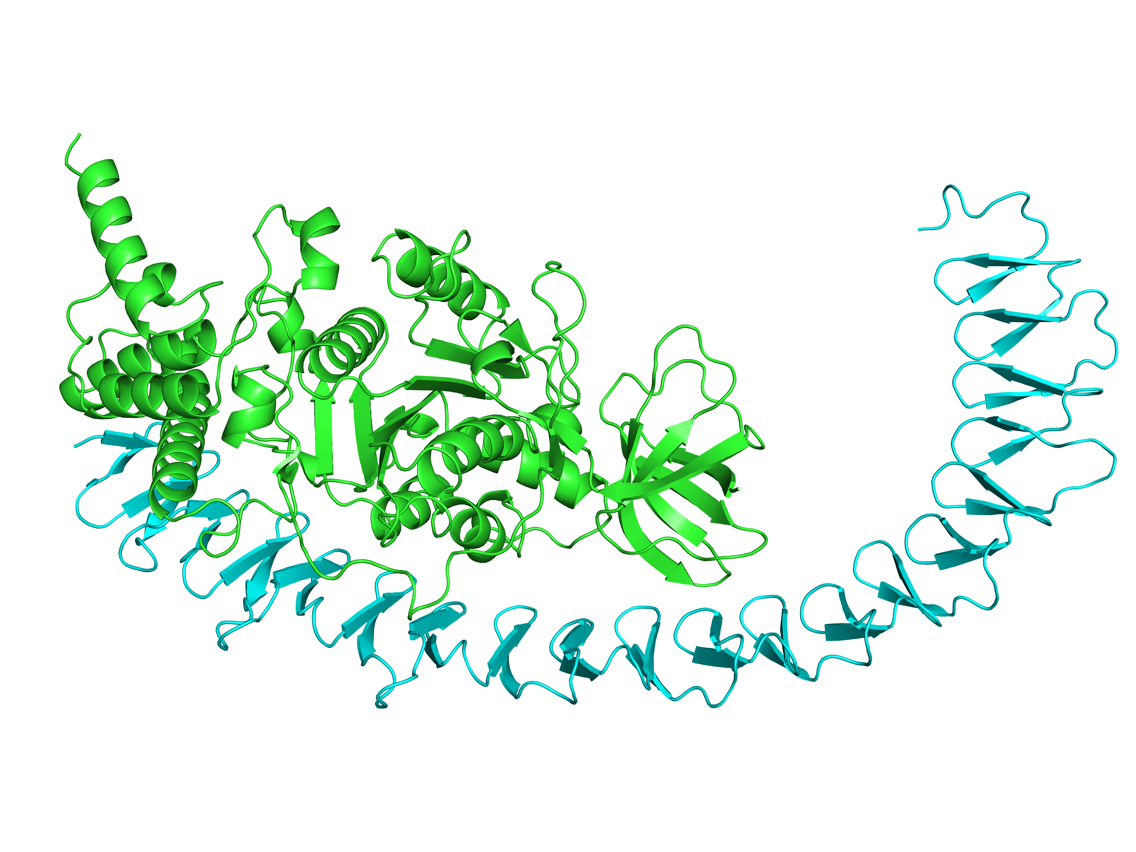
**

**Table S2: Amino acid residues involved in the interaction between galaxin and ATP5B, analyzed with MOE.**

In the "Type" column, "D" represents hydrophobic interactions (van der Waals forces), "H" indicates hydrogen bonding, "I" represents ionic interactions, and "Dist" refers to the distance between amino acids.

| **Type** | **ATP5B** | **Galaxin** | **Dist** |
| --- | --- | --- | --- |
| D | Pro138 | Leu170 | 3.98 |
| D | Pro138 | Thr181 | 3.82 |
| D | Glu139 | Leu170 | 4.13 |
| D | Glu139 | Asn182 | 3.85 |
| DH | Lys161 | Thr181 | 4 |
| DH | Lys161 | Asn182 | 3.47 |
| D | Lys161 | Thr183 | 3.69 |
| D | Lys225 | Leu142 | 3.87 |
| D | Lys225 | Tyr151 | 4.31 |
| D | Tyr230 | Ser169 | 4.25 |
| D | Ala261 | Ser169 | 3.86 |
| D | Gln296 | Tyr179 | 3.94 |
| D | Glu297 | Ala172 | 4.36 |
| D | Glu297 | Tyr179 | 4.44 |
| D | Glu297 | Thr181 | 4.2 |
| DH | Lys480 | Met111 | 3.75 |
| I | Lys485 | Asp130 | 3.84 |
| D | Glu486 | Ala113 | 4.35 |
| D | Glu486 | Cys114 | 4.05 |
| D | Glu486 | Tyr120 | 4.3 |
| DH | Lys489 | Cys96 | 4.22 |
| D | Lys489 | Cys114 | 3.88 |
| D | Glu498 | Met82 | 3.58 |
| D | His501 | Thr80 | 4.41 |
| D | His501 | Met82 | 4.09 |
| DH | Pro512 | Met122 | 3.84 |
| D | Glu514 | Ile95 | 3.94 |
| D | Glu514 | Pro112 | 3.83 |
| D | Glu514 | Alal 13 | 4.04 |
| D | Glu514 | Cys114 | 4.06 |
| D | Glu515 | Met111 | 4.06 |
| D | Val517 | Pro91 | 3.93 |
| D | Val517 | Lys104 | 3.79 |
| D | Asp521 | Leu92 | 3.66 |
| DIH | Asp521 | Lys104 | 3.73 |

**Figure S3:**

**
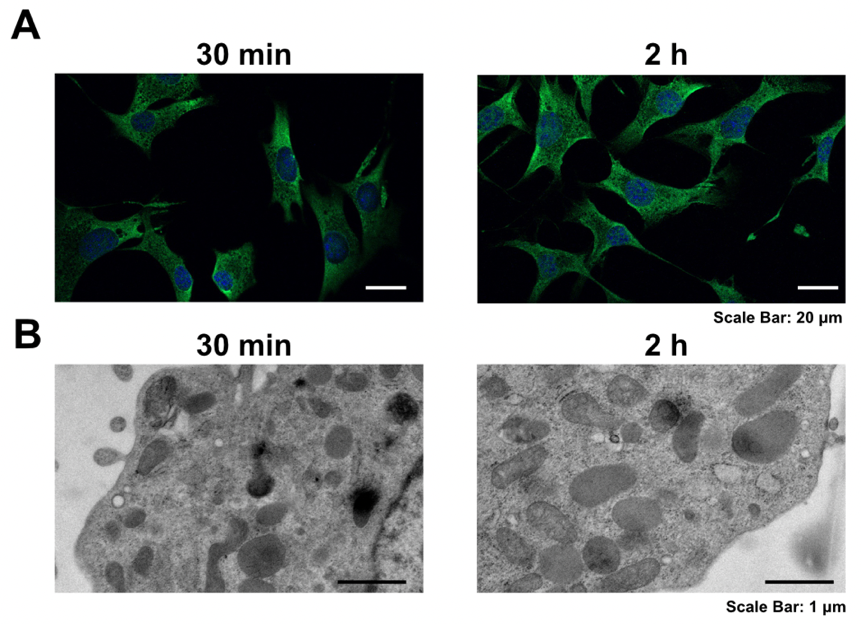
**

Figure S3: (A) Confocal microscopy experiments using his-tagged galaxin after treatment of galaxin for 30 min or 2 h, revealing intracellular localization. (B) Transmission electron microscopy (TEM) analysis after treatment of galaxin for 30 min or 2 h.
